# An Update of the Definition of Transfusion-Related Acute Lung Injury

**DOI:** 10.4274/tjh.galenos.2019.2019.0279

**Published:** 2019-11-18

**Authors:** Alexander P.J. Vlaar, Steve Kleinman

**Affiliations:** 1 Academic Medical Centre, Department of Intensive Care, Amsterdam, The Netherlands; 2 University British Columbia, Department of Pathology, Vancouver, Canada

**Keywords:** TRALI, Definition, Delphi

## To the Editor,

In the past transfusion-related acute lung injury (TRALI) was regarded as a rare complication of transfusion medicine. Subsequently, TRALI has been shown to be one of the leading causes of transfusion-related morbidity and mortality. Insight into TRALI pathogenesis in the past decades has resulted in the development of preventive strategies [1]. The accumulation of clinical and basic science knowledge has provided the rationale for a recent update of the widely used 2004 Canadian Consensus Conference (CCC) definition of TRALI (Table 1) [2]. A panel of 10 international experts on TRALI, including two members with hemovigilance expertise, used the Delphi panel approach to develop a redefinition of TRALI by modifying the 2004 CCC definition [3].

The updated TRALI definition along with the rationale for the changes has now been published (Table 2) [3]. The main modifications are as follows: 1) The term “possible TRALI” has been dropped. 2) TRALI has been separated into two types: TRALI type I (without an acute respiratory distress syndrome (ARDS) risk factor) and TRALI type II (with an ARDS risk factor or with mild preexisting ARDS). Notably, the presence of either an ARDS risk factor or mild ARDS does not exclude the diagnosis of TRALI as it did under the old definition. 3) Cases with an ARDS risk factor that meet ARDS diagnostic criteria and where respiratory deterioration over the 12 hours prior to transfusion implicates the risk factor as causative should be classified as ARDS rather than TRALI type II. 4) The 2012 updated ARDS consensus definition (referred to as the BERLIN definition) has been evaluated for its relevance to TRALI and essential updates (including guidance in diagnosing hydrostatic pulmonary edema) have been incorporated into the new TRALI definition.

More broadly, the Delphi panel recommended that all pulmonary complications after blood transfusion should be reported to the transfusion service and then categorized (either by the transfusion service, a hospital transfusion committee, or a hemovigilance system) into one of several categories: TRALI (type I or type II), ARDS, transfusion-associated circulatory overload (TACO), TRALI/TACO - cannot distinguish, or an alternate diagnosis. Importantly, the panel reaffirmed that TRALI remains a clinical diagnosis and does not require detection of cognate leukocyte antibodies, though it did recommend that these data be captured through a hemovigilance reporting system. Future research directions have been identified and include identifying the mechanism behind the onset of TRALI in the absence of cognate leukocyte antibodies. Furthermore, the panel is working on developing a universal reporting form for posttransfusion pulmonary complications including suspected TRALI.

We believe that the TRALI definition update is such an important change for transfusion medicine that it needs to be widely disseminated and discussed. To this end, the panel has submitted this letter to the editors of several important transfusion and hemovigilance journals [4,5]. We hope that the new definition contributes to an enhanced level of reporting and a more accurate classification of respiratory complications associated with blood transfusion.

## Figures and Tables

**Table 1:**
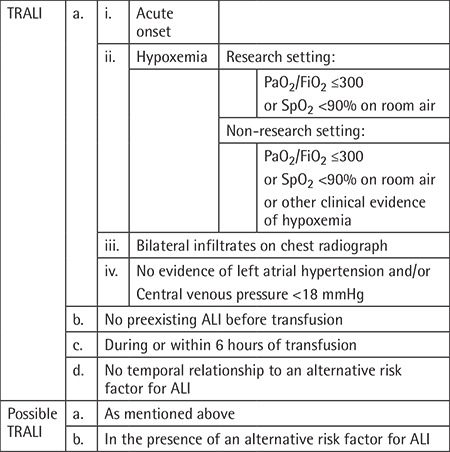
2004 Canadian Consensus Conference definition of TRALI and possible TRALI [2].

**Table 2:**
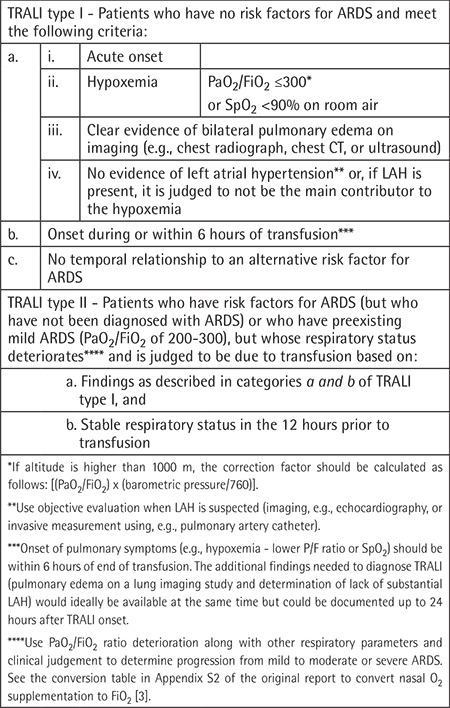
New consensus TRALI definition [3].
